# Association of Body Weight and Physical Fitness during the Elementary School Years

**DOI:** 10.3390/ijerph19063441

**Published:** 2022-03-15

**Authors:** Clemens Drenowatz, Si-Tong Chen, Armando Cocca, Gerson Ferrari, Gerhard Ruedl, Klaus Greier

**Affiliations:** 1 Division of Sport, Physical Activity and Health, University of Education Upper Austria, 4020 Linz, Austria; 2 Institute for Health and Sport, Victoria University, Melbourne 8001, Australia; sitong.chen@live.vu.edu.au; 3 Department of Sport Science, University of Innsbruck, 6020 Innbruck, Austria; armando.cocca@uibk.ac.at (A.C.); gerhard.ruedl@uibk.ac.at (G.R.); nikolaus.greier@kph-es.at (K.G.); 4 Escuela de Ciencias de la Actividad Física, El Deporte y la Salud, Universidad de Santiago de Chile (USACH), Santiago 7500618, Chile; gersonferrari08@yahoo.com.br; 5 Division of Physical Education and Sports, University of Education Stams—KPH-ES, 6422 Stams, Austria

**Keywords:** overweight, obesity, youth, cardiorespiratory fitness, muscular strength, BMI percentile, motor competence

## Abstract

Physical fitness and body weight are key correlates of health. Nevertheless, an increasing number of children display poor physical fitness and high body weight. The aim of this study was to examine the prospective association of physical fitness with body weight throughout the elementary school years with a special emphasis on children with high body weight or poor physical fitness at baseline. A total of 303 Austrian children (55.1% male) completed the German motor test up to eight times over a 4-year time span (between the ages 6 and 10 years). Physical fitness did not differ across quartiles of body weight at baseline. A more pronounced weight gain, however, was associated with an impaired development of physical fitness and this association was more pronounced in children with higher baseline body weight. In addition, the detrimental effects of an impaired development of physical fitness on subsequent body weight were more pronounced in children with higher baseline body weight. No differences in the longitudinal association between body weight and physical fitness, on the other hand, were observed across quartiles of baseline fitness. These results emphasize the importance of the promotion of physical fitness, particularly in children with increased body weight, to ensure future health.

## 1. Introduction

Excess body weight is one of the major health risks in modern society due to the association with various non-communicable diseases [1,2]. Overweight/obesity during childhood is associated with cardiovascular dysfunction and asthma [3,4,5], in addition to psychological problems including lower self-esteem, underachievement in school, and overall quality of life [6]. Children with excess body weight are also at increased risk to become overweight/obese adults [7] and, even in the absence of overweight/obesity during adulthood, children with excess body weight have an increased risk for cardiovascular disease later in life [8].

Physical fitness, which consists of cardiorespiratory endurance, muscular strength, and endurance, in addition to flexibility and body composition, is also a critical marker of health [9]. Various components of physical fitness have been associated with beneficial effects on cardiovascular and metabolic disease risk, bone health, and psychological and cognitive outcomes, which contribute to an enhanced quality of life [9,10,11,12,13]. As physical fitness is defined as a person’s ability to perform daily tasks without undue fatigue and adequate energy to enjoy leisure-time pursuits [14], it should also be considered a critical aspect in the promotion of an active lifestyle. The beneficial associations of physical fitness with various health parameters, however, are independent of physical activity [15], and children with a better physical fitness have a lower risk for metabolic and cardiovascular disease in adulthood independent of confounding factors [9,16]. Given these long-term effects, both physical fitness and body weight have been recognized as predictors of morbidity and mortality [9,10,17,18].

There is also extensive evidence on an inverse association between physical fitness and body weight [19,20,21,22], in addition to the independent association of these entities with several health outcomes. Longitudinal studies further showed that current body weight affects the development of physical fitness [23,24,25] and that poor physical fitness increases the risk for excess weight gain [23,26]. Despite considerable efforts to control excess weight gain and promote physical fitness in youth, overweight/obesity rates in children remain high and physical fitness levels have declined over the last several decades [27,28,29]. These trends are further associated with low motor competence in children, which is critical for the promotion of physical activity that, in turn, enhances physical fitness and facilitates weight management [30]. Given the long-term health implications, such a development not only affects the individual but also puts a substantial burden on the health care system [31,32,33]. Accordingly, additional actions are required to prevent the potential adverse health outcomes associated with poor physical fitness and high body weight. Recent efforts addressing low physical fitness and high body weight in children, however, have achieved limited success and it appears that a more targeted approach is warranted. This also requires a better understanding of the reciprocal association between physical fitness and body weight. The present study, therefore, examined potential differences in the cross-sectional and longitudinal association between these two entities across different levels of physical fitness and body weight in Austrian elementary school children. Given the potentially greater deficiencies in functional capacity and future health risks, a special focus was given to participants with low initial fitness and those with high body weight.

## 2. Materials and Methods

The study was conducted in the largest county of the federal state of Tyrol, Austria. Of the total 71 elementary schools in the county, 15 schools were selected via a random number generator and received information about the study. One school declined to participate due to organizational problems. The final sample, therefore, consisted of 14 schools that participated in data collection throughout the 4-year observation period. In order to track participants throughout their entire elementary school time, only students who were in first grade at baseline were eligible for participation. In addition, participants needed to be able to complete a physical fitness test battery, and children with mental, neurological, or physical diagnoses were excluded from the study. This resulted in a sample size of 392 children (55.4% male; age: 6.9 ± 0.5 years). The study protocol was approved by the Institutional Review Board of the University of Innsbruck (certificate of good standing, 16/2014), the school authorities of the federal state of Tyrol, and the school board of each participating school. Written parental consent was obtained prior to baseline data collection and children provided oral assent at the time of data collection. All study procedures were in accordance with the ethical standards of the Declaration of Helsinki (as amended in 2013).

Participants completed anthropometric measurements and physical fitness tests during each fall and spring semester over their four years in elementary school, which resulted in up to eight measurements throughout the entire observation period. Baseline data collection occurred during the school entry evaluation in October 2014 and the final follow-up measurements were completed in June 2018 when children were in their final grade (fourth grade) of elementary school. In order to be included in the analysis, participants needed to provide valid and complete data for at least five measurements, including at baseline and the last follow-up assessment.

**Figure 1:**
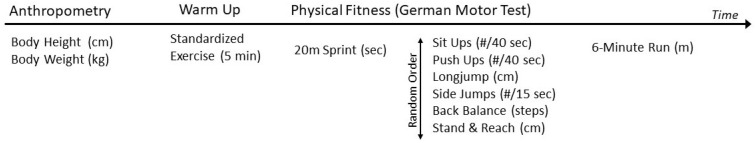
Data collection procedure at each measurement time.

Data collection occurred in the participating school’s gymnasium during regular class time in a single session. Anthropometric measurements and fitness tests were administered by exercise science graduate students, who were well trained in conducting these measurements in a pediatric population during the course of a research seminar prior to data collection. A total of 14 students were involved in the measurements throughout the 4-year study period, with 6 to 7 students present during each measurement session in the schools. An overview of the procedures for each testing session is provided in Figure 1.

Body height (cm) was measured with a portable stadiometer (SECA^®^ 217, Hamburg, Germany) and weight (kg) was measured with a calibrated digital scale (SECA^®^ 803, Hamburg, Germany) to the nearest 0.1 cm and 0.1 kg, respectively, with children wearing gym clothes and barefoot. Body mass index (BMI) was calculated (kg/m^2^) and converted to BMI percentiles (BMIPCT) using German reference values [34]. Children with a BMIPCT above the 90th percentile were classified as overweight/obese. For the statistical analyses, quartiles of baseline BMI percentiles were established (Quartile 1: BMIPCT < 29.0; Quartile 2: 29 ≤ BMIPCT < 50.2; Quartile 3: 50.2 ≤ BMIPCT < 76; Quartile 4: BMIPCT > 76.0).

Upon the completion of anthropometric measurements, participants completed the German Motor Test (DMT6-18) [35], which has been shown to provide valid and reliable information on physical fitness in children and adolescents [35,36]. The DMT6-18 consists of eight test items that assess cardiorespiratory endurance, muscular endurance, muscular strength, power, speed and agility, and balance and flexibility. Specifically, participants performed a 6 min run, sit ups, push ups, a standing long jump, a 20 m sprint, 20 s sideways jumping, backwards balancing, and a stand and reach test, with practice trials and measured attempts as specified in the test manual. Fitness tests were administered in random order after a standardized 5 min warm up, except for the 20 m sprint, which was completed at the beginning, and the 6 min run, which was completed at the end of the test session. In addition to raw performance values, the DMT6-18 provides sex- and age-standardized scores. The average of these scores is used as an indicator for overall physical fitness, with a value of 100 indicating average physical fitness for the respective age and sex; higher scores indicate above average physical fitness and lower scores indicate below average physical fitness [35]. As shown for baseline BMIPCT, quartiles for baseline physical fitness were established based on overall physical fitness scores (Quartile 1: overall physical fitness < 100; Quartile 2: 100 ≤ overall physical fitness < 105; Quartile 3: 105 ≤ overall physical fitness < 108; Quartile 4: overall physical fitness ≥ 108).

Statistical Analysis. Normal distribution of the data was confirmed prior to statistical analyses. Cross-sectional associations between BMIPCT and components of physical fitness were examined via Pearson correlation analysis. Linear mixed models (LMMs) were used to determine change in BMIPCT and overall physical fitness throughout the observation period in order to account for different time intervals between measurement periods. Subsequently, ANOVA was used to examine differences in the development of BMIPCT and physical fitness across quartiles of baseline BMIPCT and baseline physical fitness, respectively. Additionally, quantile regression analyses were performed to determine the effect of change in physical fitness and BMIPCT on physical fitness and BMIPCT at follow-up, respectively, across baseline quartiles of BMIPCT and baseline quartiles of physical fitness. In addition to change in BMIPCT or physical fitness (based on LMM), baseline BMIPCT and physical fitness were included in the regression models. Secondary analyses included sex as a co-variate to examine potential sex-specific associations. All statistical tests were performed in SPSS V26.0 software (SPSS Inc., IBM Corp., Armonk, NY, USA) with the significance level set at α < 0.05.

## 3. Results

Of the 392 eligible participants 303 children between 6 and 8 years of age at baseline (55.1% male; 9.3% overweight/obese) provided valid measurements for at least five time points, including baseline and the last follow-up measurement. There were no differences in sex distribution and anthropometric characteristics at baseline between children included in the analyses and those excluded due to missing follow-up data. Children with sufficient follow-up measurements, however, displayed better overall physical fitness at baseline compared to those excluded (104.2 ± 5.9 vs. 100.9 ± 6.5, *p* < 0.01).

Descriptive characteristics of participants included in the analyses are shown in Table 1. Boys were taller and heavier compared to girls but there was no difference in BMIPCT. Accordingly, there was also no difference in weight status and sex distribution did not differ across quartiles of BMIPCT. There was also no sex difference in overall physical fitness and sex distribution across quartiles of physical fitness. Absolute performance for the 6 min run, sit ups, standing long jump and 20 m sprint, however, was better in boys, whereas balance and flexibility was better in girls (*p* < 0.05).

Pearson correlation analyses did not show significant cross-sectional correlations between BMIPCT and components of physical fitness at baseline, except for a low inverse association between BMIPCT and 6 min run performance (Table 2). Nevertheless, the prevalence of overweight/obesity differed significantly across fitness quartiles (Q1: 4.0%, Q2: 3.0%, Q3: 1.7%, Q4: 0.7%; *p* = 0.02). However, during the last follow-up assessment, when children were 10.4 ± 0.5 years of age, significant cross-sectional associations were observed between BMIPCT and all components of physical fitness, except for flexibility. Accordingly, overall physical fitness was negatively associated with BMIPCT (r= −0.43, *p* < 0.01) when participants were in fourth grade, whereas there was no significant correlation between overall physical fitness and BMIPCT in children when they were in first grade. In addition, low significant negative correlations between weight change and change in various components of physical fitness were observed, which also resulted in a negative association of weight change with change in overall physical fitness during the elementary school years (r = −0.25, *p* < 0.01).

**Figure 2:**
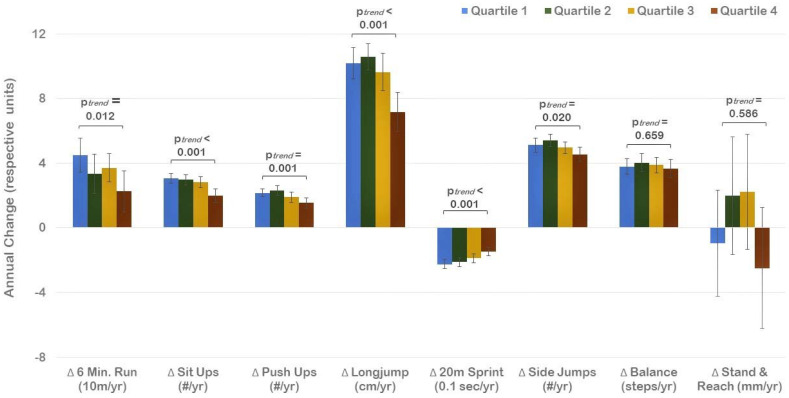
Change in physical fitness across quartiles of baseline BMI percentile (Quartile 1 indicates lowest BMI percentiles). Values are Mean with 95% CI.

**Figure 3:**
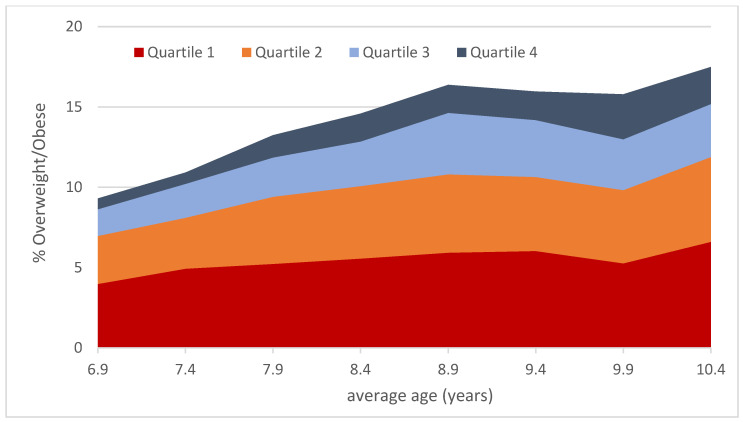
Cumulative prevalence of overweight/obesity throughout the observation period across quartiles of baseline physical fitness (Q1 indicates low physical fitness).

Longitudinal analyses showed a significant increase in the prevalence of overweight/obesity from 9.3% in first grade to 17.5% in the fourth grade. Across the entire sample, participants with a higher baseline BMIPCT displayed a lower improvement in all components of physical fitness (*p* for trend < 0.05), except for balance and flexibility (Figure 2). Change in BMIPCT, however, did not differ across quartiles of baseline BMIPCT and baseline fitness. Accordingly, the increase in the prevalence of overweight/obesity did not differ across quartiles of physical fitness (Figure 3). There were also no differences in changes in various components of physical fitness across quartiles of baseline fitness, except for the 20 m sprint (*p* for trend < 0.01), where a more pronounced improvement was detected in participants with lower baseline performance.

Linear regression analyses further showed a significant negative association between change in BMIPCT and subsequent fitness (Table 3). The detrimental effect of higher weight gain on fitness development was particularly pronounced in participants with higher BMIPCT at baseline. No clear pattern of this association was observed across baseline fitness quartiles. The association between baseline fitness and fitness at the last follow-up, however, was stronger in participants with lower baseline fitness. These results remained essentially unchanged after including sex as covariate.

Change in physical fitness was negatively associated with subsequent BMIPCT as well (Table 4). This association was also more pronounced in participants with higher baseline BMIPCT, whereas no clear pattern was observed across baseline fitness quartiles. Further, the association between baseline BMIPCT and BMIPCT at follow-up was less pronounced in participants with below-average baseline BMIPCT compared to those with higher baseline BMIPCT. The inclusion of sex as additional independent variable in the regression models did not have a significant impact on the previously reported results.

## 4. Discussion

The results of the present study show an inverse association between body weight and physical fitness in older children, whereas the association was limited in younger children. Increased body weight and low physical fitness at younger ages, however, were associated with an attenuated development of physical fitness and increased weight gain throughout the elementary school years, respectively. In addition, this study showed that the inverse reciprocal longitudinal association between body weight and physical fitness was more pronounced in children with high body weight at young ages, whereas no differences in the prospective association between body weight and physical fitness were observed across quartiles of baseline physical fitness.

These results are consistent with previous studies that showed an inverse association between body weight and physical fitness in children [20,26,37]. Musálek et al. further showed that even children who have increased body fat despite being considered normal weight displayed poorer performances on endurance and strength tests [38]. A reduction in body fat, on the other hand, was associated with an improvement in physical fitness [39]. Thus, body weight has been shown to account for a substantial portion of the variability in physical fitness during childhood [40,41] and the increase in population body weight has been associated with declines in physical fitness. Accordingly, today’s youth display lower physical fitness than those of previous generations [27,42]. The detrimental effect of increased body weight is particularly pronounced in weight-bearing activities, whereas associations between body weight and muscular strength have been less consistent [37,39,41,43,44]. This may be attributed to the fact that excess body weight may be a morphological constraint, particularly when the body needs to be moved against gravity, which potentially results in less efficient movement patterns and lower exercise tolerance, increasing the risk for withdrawal from various forms of physical activity [45,46]. Increased body weight is also associated with lower motor competence [30], which provides the foundation for engagement in various forms of physical activity [47]. The resulting lower engagement in various forms of physical activity also reduces the opportunities for the development of physical fitness [48], which results in a vicious cycle of increased body weight, poor physical fitness, and low physical activity [49]. High motor competence, on the other hand, enables children to engage in more physical activity over time, which enhances physical fitness and facilitates the maintenance of a healthy body weight. Accordingly, a recent review showed significantly higher fitness levels and lower body weight, and particularly body fat, in active compared to inactive adolescents [50]. The fact that low physical activity tracks from childhood into adolescence and adulthood [51], and increases the risk for various health problems later in life [52], further emphasizes the need for early intervention strategies.

The inverse association between body weight and physical fitness, however, appears to be limited at younger ages but strengthens with age [20,53,54]. Accordingly, change in body weight was negatively associated with the development of various aspects of physical fitness throughout the elementary school years. This, however, also implies that overweight/obese children who changed their weight status to normal weight can achieve similar fitness levels as those who were always normal weight [55]. Strategies targeting excessive fat accumulation in children, therefore, have been recommended to ensure functional capacity later in life [39]. In particular, middle childhood is considered a critical period, where positive trajectories of high physical fitness and healthy body weight or negative trajectories of poor physical fitness and increased body weight start to diverge [56]. The lack of differences in the development of body weight and physical fitness across quartiles of baseline fitness at the beginning of elementary school also indicates the high potential for the promotion of physical fitness at young ages as all children can benefit from the promotion of physical fitness, independent of their current fitness level. In particular, exercise programs of higher intensity have been shown to improve physical fitness [9,57]. Physical education in elementary school and movement programs in pre-schools, and youth sports, therefore, should be considered important intervention settings for ensuring sufficient physical fitness early in life [58]. The importance of early interventions is further emphasized by the fact that low fitness levels are sustained during adolescence, which can have major effects at the individual level and for society due to the associated health risks [39]. The results of the present study additionally highlight the importance of focusing on children with increased body weight as these have a higher risk for entering a vicious cycle of excess weight gain and poor physical fitness, which is most likely accompanied by lower physical activity levels and associated health problems.

Given the importance of physical fitness for future health among children and youth, independent of physical activity [59,60], monitoring physical fitness levels in children and adolescents has shifted from a performance-related focus to the assessment of health-related fitness [12]. At least partially due to low physical activity levels in youth, there has been a resurgence of research on health outcomes related to physical fitness in recent years [12]. Such research may be even more important in light of the observed recent declines in physical fitness due to movement restrictions in response to the COVID-19 pandemic [61]. The collection of fitness data at national and school levels, therefore, should be considered a public health priority [12]. Nevertheless, only a few countries have implemented national surveillance systems for physical fitness. One example in Europe is Slovenia, which can also be used to highlight the benefits of such efforts. Slovenia has collected physical fitness data for all children and adolescents for more than three decades via the “SLOfit” initiative [62]. In response to the observed decline in physical fitness that started in the 1990s, Slovenia implemented a national health-promotion program in 2010 that included two additional hours of physical education per week [63]. As a result, physical fitness levels have notably improved, and Slovenia was the only European country to receive an “A–“ grade for physical fitness and overall physical activity in the recent Global Physical Activity Report Card [64]. In addition to highlighting poor physical fitness in European youth, it was shown that only 9 of the 20 European countries participating in the Global Physical Activity Report Card provided adequate data to determine a grade for physical fitness in youth. This aspect further emphasizes the need for the implementation of national fitness testing initiatives that can guide the implementation of policies targeting physical fitness in youth [15].

Despite the important insights provided by the present study, there are some limitations that need to be considered when interpreting the results. There was no information on physical activity and, therefore, it was not possible to examine the association of changes in physical activity with alterations in body weight and physical fitness. The small sample size along with higher fitness levels of children that were included in the analyses compared to those excluded due to missing data may also limit generalizability of the findings. Furthermore, even though BMI is a well-accepted proxy measure for the assessment of weight status, it does not directly measure body fatness and fat distribution [65]. A higher BMI could also be associated with higher lean body mass, rather than fat mass, which has a stronger association with health compared to body weight [66]. Nevertheless, BMI is commonly used in epidemiological studies and has been shown to correlate well with body fat percentage in youth [67]. The utilization of a validated test battery that assesses various components of physical fitness, which was administered by trained personnel, on the other hand, should be considered a strength of the present study. Additionally, a total of eight measurements were administered over four years throughout the entire elementary school period, which allows for an investigation of the dynamic, prospective relationship of physical fitness and body weight. Causality, however, cannot be established due to the observational nature of this study. The insights gained, nevertheless, enhance the understanding of the complex interaction between body weight and physical fitness and, therefore, support evidence-based practice. In order to examine causal relationships, randomized controlled trials that explore effects of different exercise programs on physical fitness in youth are needed. Such longitudinal studies should also include other critical correlates of leisure time physical activity, such as self-efficacy and socio-environmental aspects, along with the assessment of health markers in order to provide further evidence on the impact of physical fitness on future health and well-being. With an increased public awareness on the influence of physical fitness and body weight on future health, there may also be a stronger commitment to emphasize the promotion of physical fitness in children as a critical contributor to public health.

## 5. Conclusions

In conclusion, the results of the present study show an inverse reciprocal relationship between body weight and physical fitness in elementary school children. The fact that this association starts to emerge during the elementary school years emphasizes the importance of early intervention strategies that minimize excess fat accumulation to ensure adequate physical fitness later in life. Additionally, it was shown that the inverse association between body weight and physical fitness was more pronounced in heavier children, whereas no differences in the progression of physical fitness were observed across different levels of baseline fitness. This highlights the potential of promoting physical fitness for each child, independent of their current fitness level. Intervention efforts, nevertheless, should pay particular attention to children with non-optimal weight status as they are at an increased risk for entering a vicious cycle of excess body weight, poor physical fitness, and low physical activity, which has a significant impact on general development and health later in life.

## Figures and Tables

**Table 1:** Descriptive characteristics at baseline for the total sample and separately for boys and girls. Values are Mean ± SD.

	Total Sample (*n* = 303)	Girls (*n* = 135)	Boys (*n* = 166)
Age (years)	6.9 ± 0.5	6.9 ± 0.5	6.9 ± 0.5
Height (cm) **	122.4 ± 5.7	121.2 ± 5.3	123.4 ± 5.9
Weight (kg) *	24.3 ± 4.4	23.6 ± 4.2	24.8 ± 4.5
BMI percentile	51.8 ± 27.3	50.2 ± 26.4	53.0 ± 28.1
6 min run (m) **	854 ± 140	823 ± 130	879 ± 143
Sit ups (# in 40 s) **	15.1 ± 5.6	14.0 + 6.0	16.0 ± 5.2
Push ups (# in 40 s)	11.6 ± 3.7	11.5 ± 3.7	11.7 ± 3.6
Long jump (cm) **	113.5 ± 17.9	106.2 ± 15.8	119.4 ± 17.3
20 m sprint (s) **	4.8 ± 0.5	5.0 ± 0.6	4.6 + 0.4
Side jumps (# in 15 s)	23.0 ± 5.8	22.3 ± 5.6	23.5 ± 5.9
Balance (steps) *	26.7 ± 9.8	28.0 ± 10.2	25.7 ± 9.5
Stand and reach (cm) ^1,^**	0.8 ± 5.6	1.9 ± 5.2	−0.1 ± 5.7
Overall fitness score (Z)	104.2 ± 5.9	103.5 ± 6.2	104.7 ± 5.6

^1^ positive values indicate reaching beyond the toes, while negative values indicate not reaching toes. * sig. sex difference (*p* < 0.05); ** sig. sex difference (*p* < 0.01).

**Table 2:** Association between body weight and physical fitness at baseline and last follow-up, and between change in body weight and change in physical fitness. Values are Pearson correlation coefficients.

		6 Min Run (m)	Sit Ups (Reps)	Push Ups (Reps)	Long-Jump (cm)	20 m Sprint (sec)	Side Jumps (Reps)	Balance (Steps)	Stand and Reach (cm)
Baseline Age: 6.9 years	BMI PCT	−0.21 **	0.02	−0.02	−0.02	−0.06	0.03	−0.06	0.12
Follow-Up Age: 10.4 years	BMI PCT	−0.36 **	−0.24 **	−0.27 **	−0.32 **	0.30 **	−0.22 **	−0.29 **	−0.01
Change (Δ 4 years)	BMI PCT	−0.15 **	−0.16 **	−0.11	−0.17 **	0.17 **	−0.13 *	−0.13 *	−0.11

* *p* < 0.05; ** *p* < 0.01; BMIPCT—BMI (body mass index) percentile; reps—repetitions in 40 s for sit ups and push ups, and repetitions in 15 s for sideways jumping.

**Table 3:** Regression coefficients based on linear regression analysis for overall physical fitness at last measurement.

	BL Fitness (β)	BL BMIPCT (β)	Δ BMIPCT (β)	R^2^
Total Sample	0.525 **	−0.309 **	−0.252 **	0.456
Low BMIPCT	0.490 **	−0.015	−0.008	0.241
<avg. BMIPCT	0.596 **	0.042	−0.238 *	0.416
>avg. BMIPCT	0.516 **	−0.126	−0.427 **	0.530
High BMIPCT	0.560 **	−0.054	−0.427 **	0.523
Low BL Fitness	0.444 **	−0.502 **	−0.211 *	0.508
Avg. BL Fitness	0.222 *	−0.287 **	−0.359 **	0.268
Above avg. BL Fitness	0.198	−0.302 **	−0.150	0.179
High BL Fitness	−0.006	0.244 *	−0.485 **	0.269

BL—baseline; BMIPCT—BMI percentile; Δ—annual change based on linear mixed model. * *p* < 0.05, ** *p* < 0.01. Low BMIPCT—BMIPCT < 29; below average BMIPCT—29 ≤ BMIPCT < 50.2; above average BMIPCT—50.2 ≤ BMIPCT < 76; high BMIPCT—BMIPCT ≥ 76. Low fitness—overall fitness < 100; average fitness—100 ≤ overall fitness < 105; above average Fitness—105 ≤ overall fitness < 108; high fitness—overall fitness ≥ 108.

**Table 4:** Regression coefficients based on linear regression analysis for overall BMI percentile at last measurement.

	BL Fitness (β)	BL BMIPCT (β)	Δ Fitness (β)	R^2^
Total Sample	−0.102 **	0.742 **	−0.203 **	0.686
Low BMIPCT	−0.144	0.235 *	−0.006	0.075
<avg. BMIPCT	−0.068	0.227 *	−0.326 **	0.155
>avg. BMIPCT	−0.289 **	0.342 **	−0.541 **	0.495
High BMIPCT	−0.104	0.372 **	−0.387 **	0.291
Low BL Fitness	0.020	0.644 **	−0.239 **	0.631
Avg. BL Fitness	−0.110	0.761 **	−0.238 **	0.740
Above avg. BL Fitness	−0.044	0.813 **	−0.101	0.733
High BL Fitness	−0.105	0.746 **	−0.261 **	0.671

BL—baseline; BMIPCT—BMI percentile; Δ—annual change based on linear mixed model. * *p* < 0.05, ** *p* < 0.01. Low BMIPCT—BMIPCT < 29; below average BMIPCT—29 ≤ BMIPCT < 50.2; above average BMIPCT—50.2 ≤ BMIPCT < 76; high BMIPCT—BMIPCT ≥ 76. Low fitness—overall fitness < 100; average fitness—100 ≤ overall fitness < 105; above average Fitness—105 ≤ overall fitness < 108; high fitness—overall fitness ≥ 108.

## Data Availability

The data presented in this study are available on request from the corresponding author.
